# Occupational radiation exposure to the lens of the eyes and its protection during endoscopic retrograde cholangiopancreatography

**DOI:** 10.1038/s41598-023-34740-5

**Published:** 2023-05-15

**Authors:** Kenji Ikezawa, Shiro Hayashi, Mamoru Takenaka, Takayuki Yakushijin, Koji Nagaike, Ryoji Takada, Takuo Yamai, Kengo Matsumoto, Masashi Yamamoto, Shunsuke Omoto, Kosuke Minaga, Shuji Ishii, Takeshi Shimizu, Kengo Nagai, Makoto Hosono, Tsutomu Nishida

**Affiliations:** 1 Department of Hepatobiliary and Pancreatic Oncology, Osaka International Cancer Institute, Osaka, Osaka Japan; 2 Department of Gastroenterology, Toyonaka Municipal Hospital, Toyonaka, Osaka Japan; 3 Department of Gastroenterology and Internal Medicine, Hayashi Clinic, Suita, Osaka Japan; 4 Department of Gastroenterology and Hepatology, Faculty of Medicine, Kindai University, Osaka-Sayama, Osaka, Japan; 5 Department of Gastroenterology and Hepatology, Osaka General Medical Center, Osaka, Osaka Japan; 6 Department of Gastroenterology and Hepatology, Suita Municipal Hospital, Suita, Osaka Japan; 7 Department of Radiology, Kindai University Faculty of Medicine, Osaka-Sayama, Osaka, Japan

**Keywords:** Biliary tract disease, Pancreatic disease, Occupational health

## Abstract

This study aimed to examine occupational radiation exposure to the lens of the eyes during endoscopic retrograde cholangiopancreatography (ERCP). In this multicenter, prospective, observational cohort study, we collected data regarding occupational radiation exposure to the lens of the eyes during ERCP. We measured radiation exposure of patients and examined its correlation with occupational exposure. In dosimetrically-measured ERCPs (n = 631), the median air kerma at the patient entrance reference point, air kerma-area product, and fluoroscopy time were 49.6 mGy, 13.5 Gycm^2^, and 10.9 min, respectively. The median estimated annual radiation dose to the lens of the eyes was 3.7, 2.2, and 2.4 mSv for operators, assistants, and nurses, respectively. Glass badge over lead aprons and eye dosimeter results were similar in operators but differed in assistants and nurses. A strong correlation was shown between eye dosimeter measurements and patients' radiation exposure. The shielding rates of the lead glasses were 44.6%, 66.3%, and 51.7% for operators, assistants, and nurses, respectively. This study revealed the actual occupational exposure dose for the lens of the eyes during ERCP and the efficacy of lead glass. Values of radiation exposure to patients can help estimate exposure to the lens of the eyes of medical staff.

## Introduction

Endoscopic retrograde cholangiopancreatography (ERCP) is a common interventional radiology (IR) procedure used to diagnose and treat pancreatobiliary diseases^1^. Since ERCP was introduced as a diagnostic procedure, the number of therapeutic applications has dramatically increased over the last two decades^2,3^. With increasing procedural complexity, awareness of occupational hazards related to radiation exposure during fluoroscopy is increasing^4,5^ for the eyes, skin, thyroid, and bone marrow, which are particularly radiosensitive and at risk of radiation-induced damage^6,7^. Although lead aprons are generally worn during ERCP, offering a certain degree of protection, the thyroid, hands, and eyes are insufficiently protected against radiation exposure in clinical practice^3,4^.

Adverse cataract events have become a concern for interventional radiologists and cardiologists^8–11^. Societies have highlighted the need for clinicians undergoing interventional procedures to consider eye protection with lead glasses^12–15^. However, the risk of radiation exposure to the lens of the eyes during ERCP remains unclear. The International Commission on Radiation Protection (ICRP) recommends a practical dose limit for eye lenses of 20 mSv in a single year or 100 mSv in any five consecutive years, without exceeding 50 mSv in a single year for occupational radiation exposure to the lens of the eyes^16,17^. Although endoscopists wear lead aprons, the use of protective eye wear is not a common practice in Japan^4^. However, there is currently little information on how much radiation endoscopists are exposed to during ERCP and the effect of lead glasses. In addition, there have been few reports evaluating the association between actual occupational radiation exposure to the lens of the eyes and radiation exposure to patients evaluated by air kerma at the patient entrance reference point (K_a,r_), air kerma-area product (P_KA_), or other parameters, such as fluoroscopy time (FT). In that context, we conducted the REX-GI (radiation exposure from gastrointestinal fluoroscopic procedures) study to prospectively collect actual radiation exposure data and to help establish national diagnostic reference levels (DRLs) for fluoroscopy-guided gastrointestinal procedures in Japan^18^. To date, there have been no reports about the relationship between occupational radiation exposure to the lens of the eyes and radiation exposure to patients during actual ERCP in prospective multicenter observational studies. In the present study, which was supplementary to the REX-GI study, we aimed to measure occupational radiation exposure to eye lenses using dosimeters attached to lead glasses during ERCP. We also examined the correlation between occupational radiation exposure to the lens of the eyes and values of radiation exposure to patients and evaluated the shielding effect of lead glasses on occupational radiation exposure to the lens of the eyes in five hospitals that were participating in the REX-GI study.

## Methods and analysis

### Study design

The REX-GI study was a multicenter, prospective, observational cohort study of radiation doses during fluoroscopy-guided gastrointestinal procedures, which was registered with the UMIN Clinical Trials Registry (UMIN000036525 (01/05/2019)) and was conducted at 23 hospitals in Japan^18,19^. We collected data regarding radiation exposure to patients (Ka,r (mGy), PKA (Gycm2), FT (min)) and radiation dose rate (RDR) (mGy/min), which was calculated as Ka,r divided by FT^19^. The present study was performed in five of 23 hospitals as part of the REX-GI study to examine occupational radiation exposure to the lens of the eyes during ERCP and the effect of lead glasses on radiation exposure to the lens of the eyes in cases where eye dosimeters were worn. The participating hospitals in the present study were Toyonaka Municipal Hospital (hospital A), Kindai University (hospital B), Osaka International Cancer Institute (hospital C), Osaka General Medical Center (hospital D), and Suita Municipal Hospital (hospital E). The fluoroscopy equipment used in the participating hospitals is shown in Table 1.

**Table 1:** Fluoroscopy equipment of participating hospitals.

	Hospital				
	A	B	C	D	E
Fluoroscopy equipment					
Company	Hitachi	Hitachi	Canon	Hitachi	Hitachi
Device	Exavista	Curevista	Ultimax-I	Curevista Versiflex	Versiflex
Apparatus type	Overtube	Overtube	Undertube	Overtube	Undertube
Year of introduction	2016	2017	2017	2018	2018
Fluoroscopy unit location	Endoscopy	Endoscopy	Endoscopy	Endoscopy	Endoscopy
Use of protective lead shields	Yes	Yes	Yes	Yes	Yes

### Dosimetry

**Figure 1:**
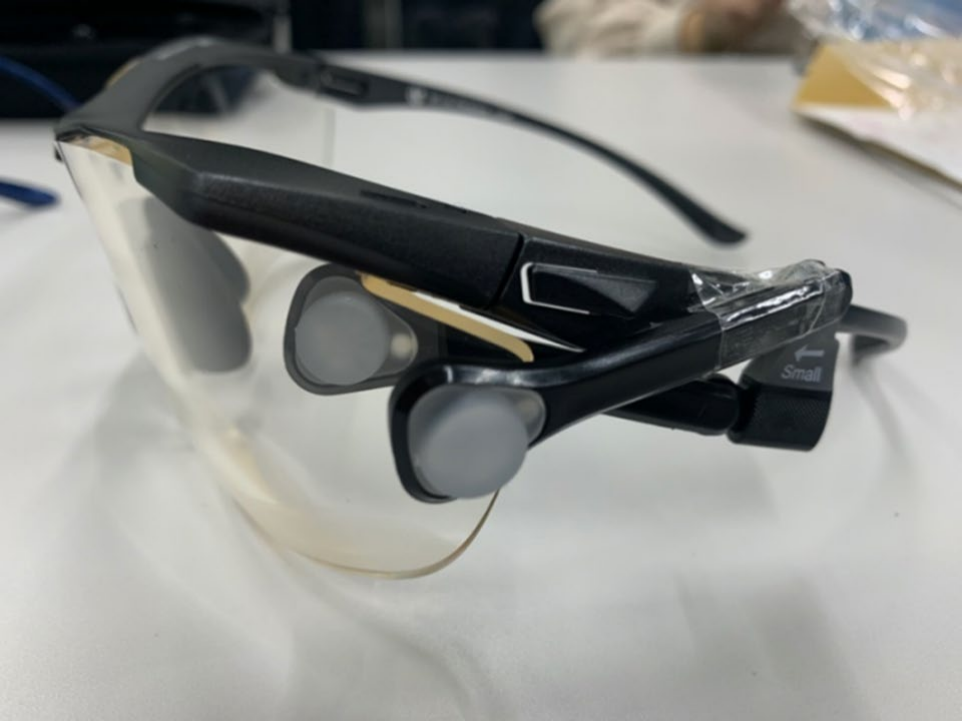
Eye dosimeter (DOSIRIS™) attached to the left side of the lead glass (inside and outside of the lead glass).

In the present study, medical staff (operator endoscopists, assistants, and nurses) wore lead glasses (0.15-mm Pb equivalent) to protect against lateral and vertical radiation (EC-10 XRAY, AOYAMAKOUGAKU, Fukui, Japan). To measure radiation exposure to the lens of the eyes (Hp (3)), an eye dosimeter, DOSIRIS™ (Chiyoda Technol Corporation, Tokyo, Japan), which is a small-sized thermoluminescent dosimeter used to estimate the 3 mm dose-equivalent (Hp (3)), was attached to the left side of the lead glass (inside and outside of the lead glass) (Fig. 1). The X-ray tube was placed on the left side of the bodies of the medical staff, although there was some movement during the procedure. Takenaka et al. previously reported that the dose to the left eye was higher than that to the right eye in operators, assistants and nurses during ERCP^20^. Therefore, we selected the left side as the measurement site for this study. The medical staff also wore glass badges (Chiyoda Technol Corporation, Tokyo, Japan) over the lead apron for Hp (10) and Hp (0.07) at the collar. They used the same dosimeter in the same position at the same hospital for three consecutive months (October 2020 to December 2020) during ERCP. Radiation doses were monitored at 1-month intervals during the study period. When the radiation dose to the dosimeters was below the measurement sensitivity, the radiation dose was treated as 0. We calculated the estimated annual radiation dose to the lens of the eyes for medical staff by multiplying the total dose for three months by 4. To examine whether glass badges worn over the lead apron can be used as a substitute for eye dosimeters, we evaluated the radiation dose measured by glass badges at the collar over the lead apron and compared it with the radiation dose measured by an eye dosimeter outside of the lead glasses. Higher values of Hp (10) and Hp (0.07) were used for the measurement results of the glass badges according to Japanese guidelines^21^.

To calculate the radiation dose per hour of fluoroscopy (mSv/hour), the radiation dose measured by an eye dosimeter outside of the lead glasses was divided by the total fluoroscopy time. We calculated the time to reach 20 mSv (eye lens dose limit per year). We also examined the correlations between occupational radiation exposure to the lens of the eyes and several values of radiation exposure to patients (Ka, r, P_KA_, and FT) to estimate the occupational radiation dose to the lens of the eyes by calculating Pearson correlation coefficients using the total values of each parameter during the study period. The coefficient of determination for the linear regression equation (R^2^) was used to assess the goodness of fit.

To calculate the shielding effect of the lead glasses, the same type of ocular dosimeter was placed on both the inside and outside of the lenses, based on previous studies. Using the obtained outer (Dout) and inner (Din) doses, the shielding rate of the glasses was calculated as follows: shielding ratio = (Dout-Din)/Dout × 100%^22^.

This study was conducted in accordance with the Declaration of Helsinki, and approval was obtained from the Institutional Review Board of Toyonaka Municipal Hospital (No. 2019-02-04, approved on April 25, 2019). Approval from Institutional Review Board was also obtained in other participating hospitals. The requirement for informed consent was waived by the opt-out method of each hospital’s website in accordance with the Ethical Guidelines for Medical Research Involving Human Subjects from the Ministry of Health, Labour and Welfare (partially modified on April 7, 2017). A waiver of informed consent was granted by institutional review board of the Institutional Review Board of Toyonaka Municipal Hospital.

### Data analysis

Data are expressed as numbers and percentages for categorical variables and as medians with interquartile ranges (IQRs) for continuous variables. All statistical analyses were performed using EZR (Saitama Medical Center, Jichi Medical University, Saitama, Japan) (ver. 1.54; R Foundation for Statistical Computing, Vienna, Austria)^23^ and JMP software (ver. 15.2.0, SAS Institute, Inc., Cary, NC, USA). For all tests, a P value of < 0.05 was considered statistically significant.

## Results

### Patient characteristics and radiation exposure to patients

Out of a total of 709 ERCPs that were performed at five institutions during the study period, 631 ERCPs were dosimetrically measured (23–217 per institution). The patient characteristics (age, sex, and disease site) are shown in Table 2. For radiation exposure to patients, the median K_a,r_ was 49.6 mGy (IQR: 27.9–105.5 mGy), the median PKA was 13.5 Gycm^2^ (7.0–25.0 Gycm^2^), the median FT was 10.9 min (6.0–19.0 min), and the median RDR was 5.0 mGy/min (IQR: 3.7–6.3 mGy/min). Data regarding the radiation exposure to patients at each hospital are shown in Table 2.

**Table 2:** Patient characteristics and radiation exposure to patients.

	Hospital					
	All hospitals	A	B	C	D	E
Patients						
Total number	631	112	217	105	174	23
Age, median (IQR)	73 (66–81)	76 (69–81)	74 (66–81)	71 (62–74)	75 (69–83.75)	75 (70–80.5)
Sex, male: Number (%)	383 (60.7)	74 (66.1)	123 (56.7)	71 (67.6)	101 (58.0)	14 (60.9)
ERCP						
Disease sites						
Common bile duct stone	217	58	94	1	54	10
Proximal malignant biliary obstruction	91	9	31	26	22	3
Distal malignant biliary obstruction	172	20	46	66	31	9
Pancreatic disease	63	6	25	1	31	0
Others	88	19	21	11	36	1
Radiation exposure to patients						
K_a,r_ (mGy), median (IQR)	49.6 (27.9–105.5)	34.7 (20.6–58.9)	55.9 (29.0–139.5)	48.0 (29.0–111.0)	53.5 (30.0–103.5)	65.1 (50.2–84.8)
P_KA_ (Gycm^2^), median (IQR)	13.5 (7.0–25.0)	7.1 (4.3–11.8)	17.7 (8.3–30.0)	15.0 (10.0–27.0)	12.0 (7.0–22.0)	24.6 (17.6–31.3)
FT (min), median (IQR)	10.9 (6.0–19.0)	9.0 (5.0–15.5)	12.2 (7.5–21.7)	9.9 (6.2–17.7)	10.0 (6.0–20.8)	12.0 (9.0–14.2)
RDR (mGy/min), median (IQR)	5.0 (3.7–6.3)	3.7 (2.8–6.1)	4.9 (3.6–6.5)	4.8 (3.7–6.8)	5.4 (4.3–6.1)	6.1 (4.9–6.9)

IQR, interquartile range; ERCP, endoscopic retrograde cholangiopancreatography; K_a,r_, air kerma at the patient entrance reference point; P_KA_, air kerma-area product; FT, fluoroscopy time; and RDR, radiation dose rate, which was calculated as K_a,r_ divided by FT.

### Annual occupational radiation exposure to the lens of the eyes and comparison with measurement results of glass badges at the collar

To predict occupational radiation exposure to the lens of the eyes when lead glasses were not worn, we evaluated the lens dose measured by an eye dosimeter outside of the lead glasses. In operators, the median annual lens dose measured by an eye dosimeter outside the lead glasses was 3.7 mSv (IQR: 2.0–7.3 mSv). The annual lens dose measured by an eye dosimeter outside of the lead glasses was smaller in assistants [median 2.2 mSv (IQR: 1.0–2.3 mSv)] and nurses [median 2.4 mSv (IQR: 2.2–2.8 mSv)] (Table 3).

**Table 3:** Annual radiation exposure to the lens of the eyes measured by eye dosimeter outside of lead glasses and comparison with measurement results of glass badges.

	All	Hospital				
		A	B	C	D	E
ERCP procedures	631	112	217	105	174	23
Annual radiation dose measured by eye dosimeter outside of lead glasses	Median, (IQR)					
Operator (mSv)	3.7 (2.0–7.3)	3.7	19.6	2.0	7.3	1.2
Assistant (mSv)	2.2 (1.0–2.3)	1	6.1	2.2	2.3	0
Nurse (mSv)	2.4 (2.2–2.8)	2.2	4.0	2.4	2.8	2.0
Annual dose calculated by measurement results of glass badges						
Operator (mSv)	3.6 (2.8–4.4)	3.6	9.6	2.8	4.4	1.6
Assistant (mSv)	1.2 (0.4–1.6)	0.4	2.8	1.6	1.2	0
Nurse (mSv)	1.6 (1.2–2.4)	1.2	2.4	1.2	2.8	1.6
Gap from radiation dose measured by eye dosimeters to measurement results of glass badges						
Operator (%)	− 2.2 (− 39.6 to 37.3)	− 2.2	− 51.1	37.3	− 39.6	37.9
Assistant (%)	− 51.1 (− 55.5 to 43.0)	− 60.0	− 53.9	− 27.3	− 48.3	NA
Nurse (%)	− 39.4 (− 46.4 to 20.0)	− 46.4	− 39.4	− 50.0	− 1.4	− 20.0
*Radiation dose per hour of fluoroscopy*						
Operator (mSv/h)	0.041 (0.020–0.044)	0.041	0.087	0.020	0.044	0.058
Assistant (mSv/h)	0.014 (0.011–0.022)	0.011	0.027	0.022	0.014	0
Nurse (mSv/h)	0.024 (0.018–0.025)	0.025	0.018	0.024	0.017	0.100
Estimated time to reach 20 mSv						
Operator (h)	457.1 (345.5–484.4)	484.4	229.8	981.9	457.1	345.5
Assistant (h)	1172.5 (868.4–1521.5)	1782.7	742.3	910.5	1434.5	NA
Nurse (h)	834.6 (795.8–1139.6)	795.8	1139.6	834.6	1171.8	200.4

IQR, interquartile range; ERCP, endoscopic retrograde cholangiopancreatography; NA, not available.

To predict whether the measurement results of glass badges worn over lead aprons can be used as a substitute for eye dosimeters, we compared them with the radiation dose measured by an eye dosimeter outside of the lead glasses. The annual dose calculated by the measurement results of glass badges was 3.6 mSv (IQR: 2.8–4.4 mSv) for operators, 1.2 mSv (IQR: 0.4–1.6 mSv) for assistants, and 1.6 mSv (IQR: 1.2–2.4 mSv) for nurses. For operators, the median difference from the radiation dose measured by the eye dosimeter to the measurement results of the glass badge was − 2.2% (IQR: − 39.6 to 37.3%) (Table 3). For assistants and nurses, the median difference was large (assistants: median − 51.1% (IQR: − 55.5 to 43.0%); nurses: median − 36.4% (IQR: − 46.4 to 20.0%)).

### The correlation between occupational radiation exposure to the lens of the eyes and radiation exposure to patients

Next, we examined the radiation dose per fluoroscopy time (Table 3). The median radiation dose per hour of fluoroscopy was 0.041 mSv/hour (IQR: 0.020–0.044 mSv/hour) for operators, 0.014 mSv/hour (IQR: 0.011–0.022 mSv/hour) for assistants, and 0.024 mSv/hour (IQR: 0.018–0.025 mSv/hour) for nurses. The median estimated time to 20 mSv (eye lens dose limit per year) was 457.1 h (IQR: 345.5–484.4 h) for operators, 1172.5 h (IQR: 868.4–1521.5 h) for assistants, and 834.6 h (IQR: 795.8–1139.6 h) for nurses (Table 3).

**Figure 2:**
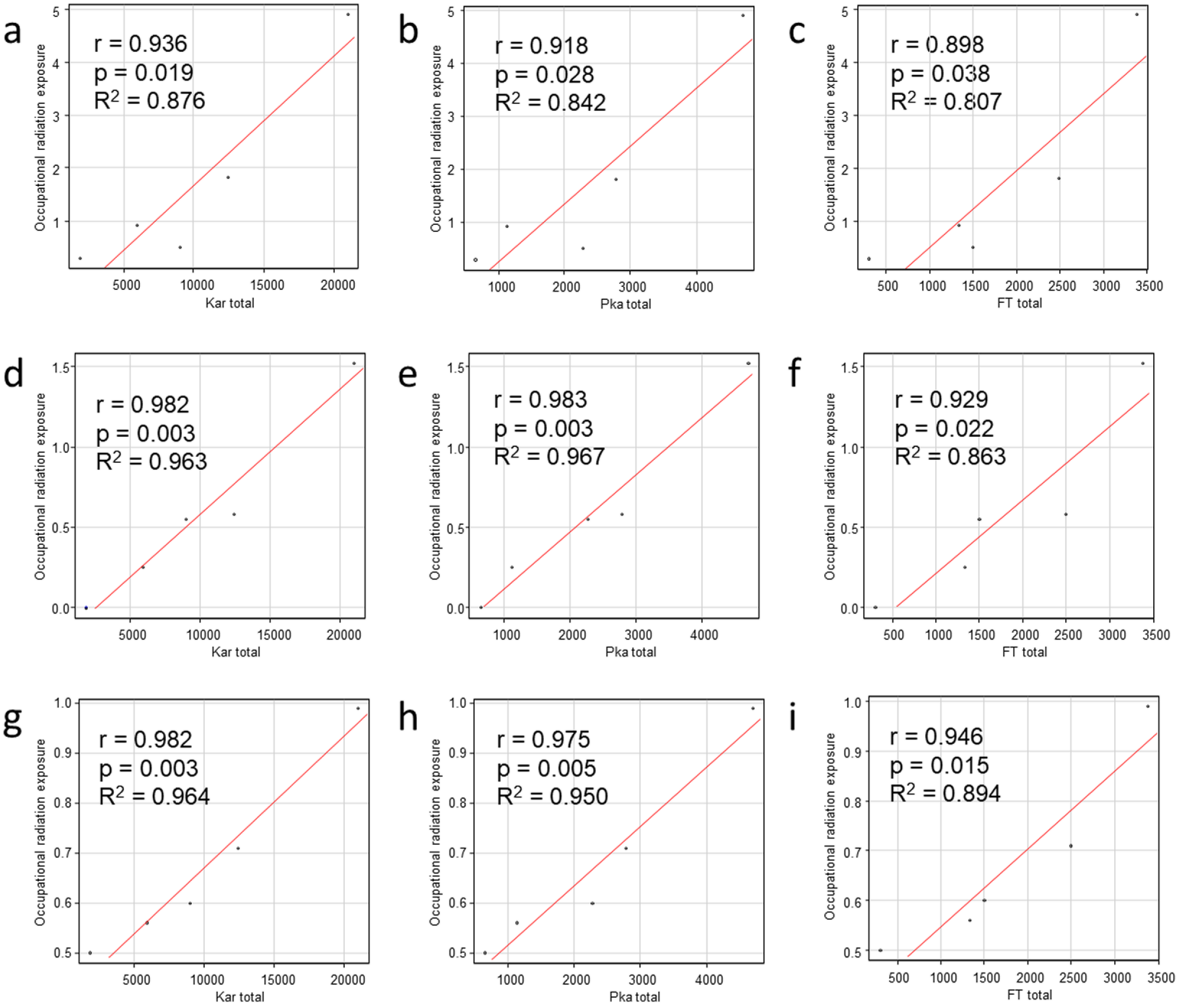
Correlations between occupational radiation exposure to the lens of the eyes (mSv) and radiation exposure to patients (air kerma at the patient entrance reference point (K_a,r_: mGy), air kerma-area product (P_KA_; Gycm^2^), fluoroscopy time (FT; min)). r: correlation coefficient, p: probability value of the correlation coefficient, R^2^: coefficient of determination. **a–c** Radiation exposure to the lens of the eyes of operators and radiation exposure to patients (**a** K_a,r_, **b** P_KA_, **c** FT). 2d-f: Radiation exposure to the lens of the eyes of assistants and radiation exposure to patients (**d** K_a,r_, **e** P_KA_, **f** FT). **g**–**i** Radiation exposure to the lens of the eyes of nurses and radiation exposure to patients (**g** K_a,r_, **h** P_KA_, **i** FT).

We also examined the correlation between occupational radiation exposure to the lens of the eyes and values of radiation exposure to patients (K_a,r_, P_KA_, and FT) to examine whether occupational radiation exposure to the lens of the eyes can be predicted by values of radiation exposure to patients. For operators, assistants and nurses, Pearson correlation analysis revealed strong, positive correlations between occupational lens exposure and radiation exposure to patients (K_a,r_, P_KA_, and FT) (operators: K_a,r_, r = 0.936, *p* = 0.019, R^2^ = 0.876; P_KA_, r = 0.918, *p* = 0.028, R^2^ = 0.842; FT, r = 0.898, *p* = 0.038, R^2^ = 0.807; assistants: K_a,r_, r = 0.982, *p* = 0.003, R^2^ = 0.963; P_KA_, r = 0.983, *p* = 0.003, R^2^ = 0.967; FT, r = 0.929, *p* = 0.022, R^2^ = 0.863; nurses: K_a,r_, r = 0.982, *p* = 0.003, R^2^ = 0.964; P_KA_, r = 0.975, *p* = 0.005, R^2^ = 0.950; FT, r = 0.946, *p* = 0.015, R^2^ = 0.894) (Fig. 2).

### Shielding effect of lead glasses on occupational radiation exposure to the lens of the eyes

Finally, we examined the shielding effect of lead glasses by comparing the radiation dose measured by an eye dosimeter inside of the lead glasses with that outside of the glasses. The median radiation dose measured by the eye dosimeter inside of lead glasses was 2.4 mSv (IQR: 1.1–5.3 mSv) for operators, 0.4 mSv (IQR: 0–1.5 mSv) for assistants, and 1.2 mSv (IQR: 1.0–2.4 mSv) for nurses (Table 4). The shielding rate of the lead glasses was 44.6% (IQR: 35.9–45.1%) for operators, 66.3% (IQR: 48.5–85.0%) for assistants, and 51.7% (IQR: 16.9–57.1%) for nurses.

**Table 4:** Shielding effect of occupational radiation exposure to the lens of the eyes with lead glass.

	All	Hospital				
		A	B	C	D	E
ERCP procedures	631	112	217	105	174	23
Annual radiation dose measured by eye dosimeter	Median, (IQR)					
Outside of lead glasses						
Operator (mSv)	3.7 (2.0–7.3)	3.7	19.6	2.0	7.3	1.2
Assistant (mSv)	2.2 (1.0–2.3)	1	6.1	2.2	2.3	0
Nurse (mSv)	2.4 (2.2–2.8)	2.2	4.0	2.4	2.8	2.0
Inside of lead glasses						
Operator (mSv)	2.4 (1.1–5.3)	2.4	10.9	1.1	5.3	0
Assistant (mSv)	0.4 (0–1.5)	0	2.9	0.4	1.5	0
Nurse (mSv)	1.2 (1.0–2.4)	1.0	3.7	1.2	2.4	0.5
Shielding rate of the lead glasses						
Operator (%)	44.6 (35.9–45.1)	35.9	44.6	45.1	26.9	100
Assistant (%)	66.3 (48.5–85.0)	100	52.6	80.0	36.2	NA
Nurse (%)	51.7 (16.9–57.1)	57.1	6.1	51.7	16.9	74.0

IQR, interquartile range; ERCP, endoscopic retrograde cholangiopancreatography. NA, not available.

## Discussion

Radiation exposure to the lens of the eyes is one of the most critical problems physicians face during medical fluoroscopic procedures^24,25^. In digestive endoscopy, medical radiation is used in various endoscopic procedures, and ERCP is the most common procedure performed under fluoroscopic guidance^26^. However, few studies have evaluated actual radiation to the lens of the eyes during ERCP using a specific dosimeter for radiation exposure to the lens of the eyes^20,27^. This study revealed that radiation exposure to the lens of the eyes for Hp (3) while wearing the eye dosimeter DOSIRIS™ just lateral to the eyes was a median estimated annual lens dose of 3.7 mSv for operator endoscopists, measured at the shortest distance from the fluoroscopy table. In addition, it was suggested that the estimated annual radiation exposure to the lens of the eyes in the high-volume hospitals of ERCPs may have reached approximately 20 mSv. Although the number of ERCP procedures varied among hospitals, we analyzed the radiation dose per hour of fluoroscopy. This revealed that the median time to reach 20 mSv per year was 457 h (the shortest was 230 h). While there have been estimates of radiation exposure to the lens of the eyes for medical personnel using phantom models^28,29^, there have been few reports examining its relationship with fluoroscopy time, which is considered valuable^27^. Although it is unlikely that a single endoscopist would reach this time for 20 mSv, the significant differences observed between the hospitals suggest that radiation dose for the lens of the eyes is highly dependent on the facility environment. Therefore, it is important to know the amount of radiation exposure to the lens of the eyes received at each hospital. Although studies where dosimeters are attached to lead glasses during interventional radiology are gradually increasing, eye dosimeters have not been widely used in daily practice because they require additional time, cost and effort^30,31^. Therefore, it is important to examine whether body dosimeter exposure can represent radiation exposure to the lens of the eyes. The present study showed that the measurement results of glass badges worn over lead aprons were similar to the radiation dose measured by the eye dosimeter in operators, while they largely differed in assistants and nurses. Moreover, there was a large variation in the measured values among the hospitals. These results suggest that the measurement results of glass badges worn over lead aprons are not enough to be used as a substitute for eye dosimeters.

In this study, we found a strong, positive correlation between occupational radiation exposure to the lens of the eyes and values of radiation exposure to patients. These results suggest that the radiation exposure values of patients can be used to estimate occupational radiation exposure to the lens of the eyes. Therefore, even if radiation exposure to the lens of the eyes is not directly measured by eye dosimeters, we can reduce it by being aware of DRLs and the facility's exposure values because they are proportional to occupational radiation exposure to the lens of the eyes. Consequently, medical radiation exposure control using DRLs is becoming more important.

In addition, we evaluated the shielding effects of lead glasses for radiation protection during ERCP and found that their shielding effects were 45%, 66%, and 52% for operators, assistants, and nurses, respectively. Differences in shielding rates by occupation may be influenced by the positional relationship to the irradiator and movement of medical staff during procedures. These results were similar to those reported in other experimental studies^28^, which showed that lead glasses decreased eye lens exposure by approximately 50%. However, because verification during actual ERCP is rarely reported^20^, the fact that the shielding effect of lead glasses could be expected during ERCP is also considered a major strength of this study. Based on these results, we recommend the use of lead glasses during ERCP.

This study has several limitations. First, in this study, dosimeters were not worn on an individual basis but were shared by each operator, assistant, or nurse in consecutive ERCP procedures at each facility. This made it difficult to assess individual differences in medical radiation exposure but allowed us to identify differences between facilities. Second, the annual exposure was estimated from three months of data, which may not accurately represent annual exposure. However, the evaluation was conducted using coherent and continuous ERCP for three months, and we believe that the difference would be small. Finally, there were two types of fluoroscopy equipment used for ERCP in this study: over-tube (3 hospitals) and under-tube (2 hospitals). Radiation protection should be considered for each type because the scattered dose distribution is different. Due to small number of hospitals, however, we did not compare types of fluoroscopy equipment in this study.

## Conclusion

This prospective study revealed the actual radiation exposure to the lens of the eyes of medical staff during ERCP, positive and strong correlations between occupational radiation exposure to the lens of the eyes and values of radiation exposure to patients (K_a,r_, P_KA_, and FT), and efficacy of lead glasses for decreasing radiation exposure to the lens of the eyes. Although aggressive use of eye dosimeters is recommended for accurate measurement, values of radiation exposure to patients can help in estimating radiation exposure to the lens of the eyes of medical staff because of their strong correlation.

## Data Availability

The data that support the findings of this study are available upon request from the corresponding author.
